# Pseudoendoleak: A post‐endovascular aortic repair ultrasound surveillance pitfall – Single institution case series

**DOI:** 10.1111/1754-9485.13409

**Published:** 2022-04-10

**Authors:** James P Lisik, Greg Curry, Timothy Buckenham

**Affiliations:** ^1^ Department of Diagnostic Imaging Monash Health Clayton Victoria Australia; ^2^ Christchurch Clinical School of Medicine University of Otago Otago New Zealand; ^3^ Department of Diagnostic Imaging Christchurch Hospital Christchurch New Zealand

**Keywords:** contrast‐enhanced ultrasound, endoleak, endovascular aortic repair, EVAR, pseudoendoleak

## Abstract

**Introduction:**

In the context of increasingly common endovascular treatment for abdominal aortic aneurysms, endoleak is a relatively common complication of (abdominal) EVAR, and ongoing multimodality surveillance programs are recommended by expert bodies including the Society for Vascular Surgery (SVS). We describe the colour doppler ultrasound (CDUS) finding defined as pseudoendoleak that may be misinterpreted as significant endoleak and may be resolved through the use of contrast‐enhanced ultrasound (CEUS).

**Methods:**

Retrospective review of cases at our institution identified five cases where apparent endoleak on CDUS was not evident on CEUS, performed immediately following CDUS.

**Results:**

Each of these five cases demonstrated interval increase in sac size at varying intervals post‐EVAR, and in 4 out of 5 cases, no endoleak was demonstrated on multiple other modalities, at multiple time points. One case demonstrated an isolated type 2 endoleak at one time point, a finding that could not be reproduced. In each case, index‐positive CDUS is thought to represent agitated fluid within the excluded sac that is not in continuity with the arterial blood pool as evidenced by the absence of CEUS enhancement.

**Conclusions:**

In cases of positive post‐EVAR CDUS, CEUS is an effective tool to exclude the presence of pseudoendoleak and thus avoid further and potentially invasive diagnostic modalities in an elderly and comorbid cohort.

## Introduction

Pseudoendoleak is here defined as the presence of Doppler shift seen on colour doppler ultrasound (CDUS) emanating from the agitation of fluid contained within the excluded sac following abdominal endovascular aortic repair (EVAR) graft pulsation without evident arterial communication as proven by a negative contrast‐enhanced ultrasound (CEUS).

### Background

Abdominal aortic aneurysms (AAAs) are a common manifestation of cardiovascular disease, with an overall prevalence of 4%; 7.6% in men aged 65–80.^1^ Aneurysm repair, surgical or endovascular, is considered for patients with >50‐mm AAA or interval aneurysmal sac expansion. The annualised risk of mortality increases exponentially with sac sizes >5–6 cm.^2^ Similar outcomes are seen with endovascular and open repair, with decreased in‐hospital mortality but increased reintervention rates associated with endovascular aortic repair (EVAR).^3^

A number of different imaging modalities may be used for post‐EVAR surveillance, primarily CTA and CDUS. Magnetic resonance imaging (MRI) and digital subtraction angiography (DSA) are rarely used.^4^, ^5^ Computed tomography angiography (CTA) and CDUS assess changes in aneurysm sac size and for the presence of endoleaks. While literature demonstrates decreased sensitivity of CDUS compared with CTA, there are distinct advantages of ultrasound with a lack of ionising radiation, potentially nephrotoxic contrast and improved temporal resolution.^6^ The typical schedule at our institution (assuming an uncomplicated course) would involve baseline studies approximately 1‐month postintervention, followed by annual assessments, typically with CDUS, in line with SVS guidelines.^7^ In the event of increasing sac size or findings suggesting endoleak, further imaging would be performed.

Contrast‐enhanced ultrasound has the advantage over CT of incorporating time‐resolved assessment and its use in differentiating Type 1 and Type 2 endoleak based on this property is described.^4^ Indeed, in many studies, it is shown to have a higher sensitivity than CTA.^8^, ^9^ CEUS is used at our institution in cases where there is sac expansion with no endoleak seen on CT or CDUS, given its improved sensitivity.^4^, ^10^ Of note, in Australia, CEUS is not currently Medicare rebateable for post‐EVAR imaging.

### Aims

While several papers describe false positives on CDUS when compared against a standard of CTA or DSA,^11^ to the authors' knowledge, there is little if any published literature on the utility of CEUS in differentiating pseudoendoleak (i.e. a form of false positive) from a true endoleak. We present a case series describing such use of CEUS at our institution.

## Methods

### Ethics

This project was granted QA (quality assurance) ethics approval by the Monash Health Human Research Ethics Committee.

### Case search method

The local radiology information system (RIS) database was searched for all contrast‐enhanced ultrasound studies performed between January 2014 and September 2021. A combination of searches using multiple examination codes and specific search terms was used to ensure that all relevant studies were captured. Searches were combined into an Excel spreadsheet and filtered. Duplicates were excluded and each study was carefully cross‐referenced with the picture archiving system (PACS) to ensure they met the inclusion criteria of expanding sac with a negative CT, positive CDUS with a negative CT, positive CT with uncertainty as to the source of endoleak. Seventy‐seven examinations where CEUS was performed for post‐EVAR assessment of endoleak were identified. Each was reviewed, with 5 cases meeting inclusion criteria.

#### US protocol

##### Patient preparation

Patients are asked to fast for a minimum of 4 h prior and positioned supine for the examination.

##### Pre‐CEUS assessment

Prior to administering contrast agent, a full assessment of endograft is performed. If fenestrations are present, then these are also assessed. The aneurysm sac is measured and cross‐referenced against available previous imaging and then assessed for evidence of endoleak. The use of a combination of CDUS, PDUS (power Doppler ultrasound) and minimal flow imaging (MFI) is undertaken with careful optimisation of low flow settings. When flow is seen in the excluded sac, identification of the source is attempted with the use of spectral Doppler analysis.

##### *Contrast—*(DEFINITY **^®^**)

*A c*ontraindication checklist is routinely performed. Contrast is agitated as per the manufacturer's guidelines using VIALMIX^®^.

1.5 mL of agitated Definity contrast media is diluted to 10 mL with 8.5 mL of NaCl 0.9%. Contrast settings on the ultrasound system are set up with side‐by‐side reference images and optimised prior to injection. A 3‐mL aliquot of the solution is injected and flushed with saline, and the contrast timer is started.

The aneurysm and endograft are insonated over several minutes to assess for evidence of contrast media in the sac. Destruction‐reperfusion technique is used when required to assess the speed of contrast re‐accumulation and/or differentiate bubbles from artefacts.

Subsequent aliquots up to a total of 10 ml of solution can be injected as required for further assessment. In cases where no contrast media are visualised in the sac, then a minimum postcontrast scan time of 5 min with multiple injections is performed to exclude late‐ or slow‐filling endoleaks.

### Cases

#### Case 1

**Fig. 1:**
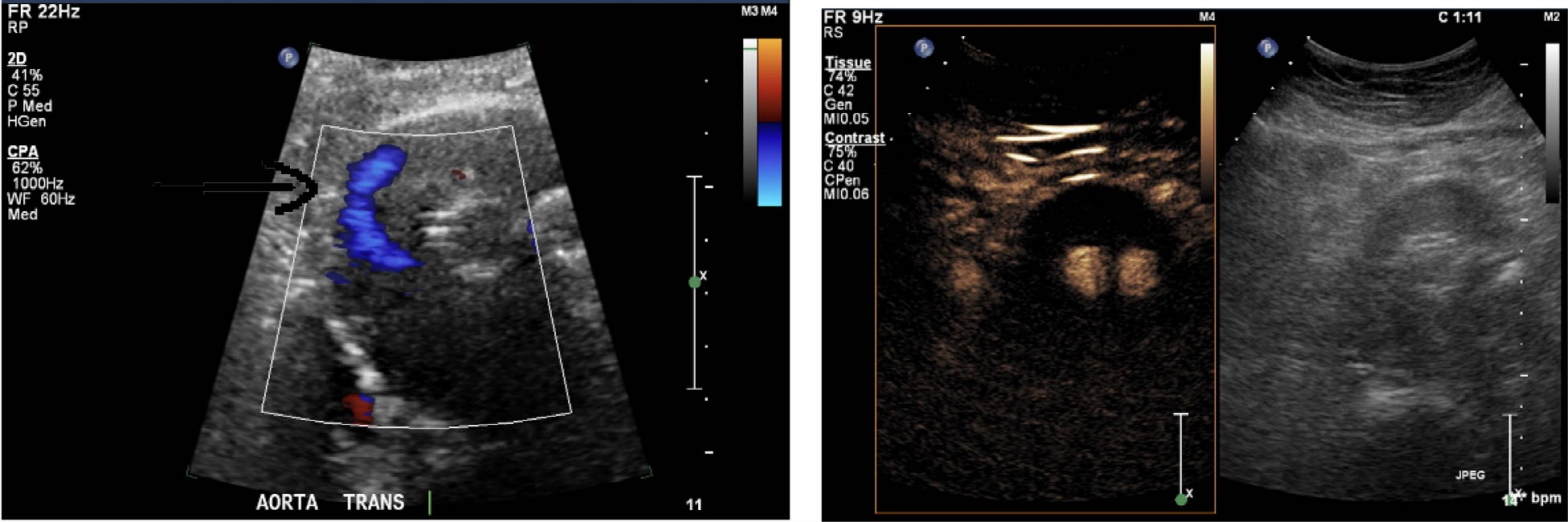
CDUS (left) showing doppler flow within the excluded sac; CEUS (right) without demonstrable endoleak.

An 86‐year‐old man at the time of index ultrasound was referred for sonographic evaluation 22 months following uncomplicated EVAR, with a marginal increase in sac size from 64 x 62 mm on index postoperative CT to 67 × 67 mm 12 months later. CDUS demonstrated flow within the sac but no endoleak on CEUS (see Fig. 1).

Subsequent CT‐guided sac puncture and DSA did not demonstrate endoleak with serous fluid aspirated and arterial pressure transduced at sac puncture. The graft was relined 4 weeks later for treatment of SEWEL without further expansion.

#### Case 2

A 75‐year‐old man at the time of index ultrasound was referred for increasing sac size (50 x 46 mm to 70 × 65 mm) on CTA over a period of 4 years without demonstrable endoleak. The first available imaging was 11‐month post‐EVAR and the EVAR itself 5 years prior to index ultrasound.

**Fig. 2:**
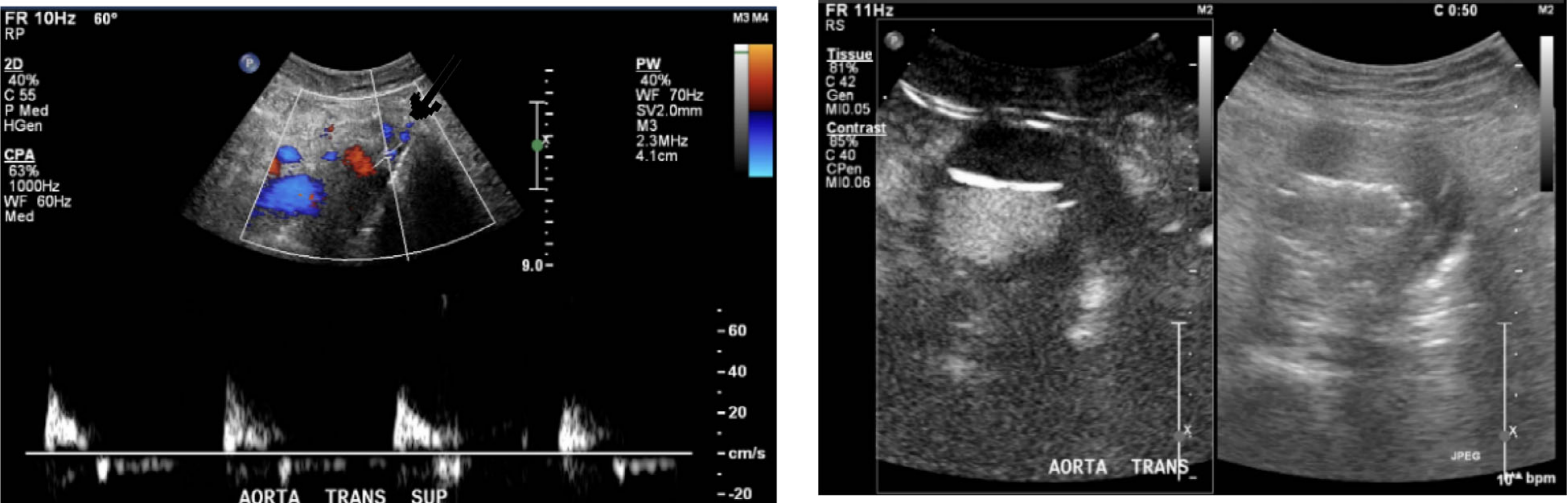
CDUS (left) showing flow within the excluded sac, with pulsatile movement within the sac; CEUS (right) without demonstrable endoleak.

CDUS demonstrated pulsatile flow within the sac but no endoleaks on CEUS (see Fig. 2).

Two subsequent CTA over the course of 3 months also failed to demonstrate any source of endoleak and demonstrated a stable sac diameter. An open repair was performed 4 months after index ultrasound, with serous fluid noted within the aneurysm sac at surgery.

#### Case 3

A 77‐year‐old man at the time of index ultrasound was referred for sac expansion. Review of prior imaging demonstrated a sac size of 49 × 52 mm, with increase to 56 x 59 mm over 6 years (on CT). His original EVAR was 7 years earlier.

**Fig. 3:**
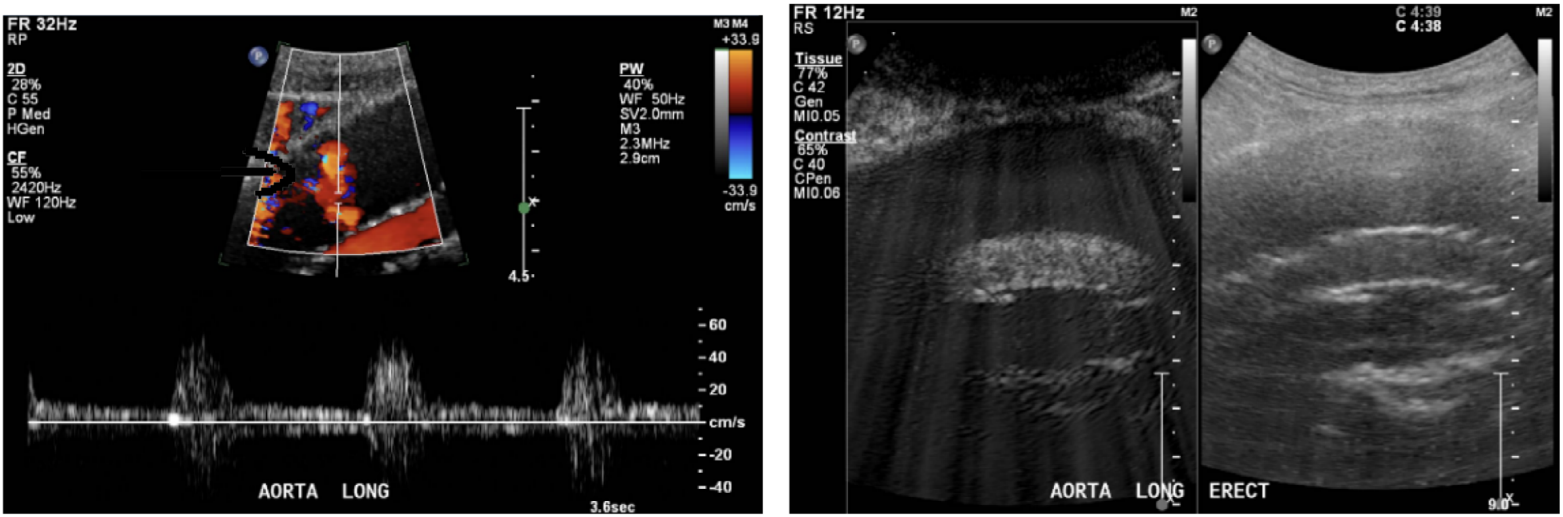
CDUS (left) showing pulsatile flow within the excluded sac; CEUS (right) without demonstrable endoleak (sagittal plane).

Index CDUS demonstrated flow within the sac, thought to be emanating from the anterior aspect of the graft body. CEUS was negative for endoleak (see Fig. 3).

These findings were confirmed on repeat CEUS 17 months later, though an increase in maximal sac diameter from 58 mm to 62 mm was noted. Follow‐up was recommended in the context of SEWEL; however, the patient died of an unrelated cause prior to follow‐up (Table 1).

**Table 1:** Demographics and key information

	Mean	Range
Age at index US (y)	81.4	75–89
Male	100%	n/a
Maximal sac size increase (mm)*	4.2	1–7
Years post‐EVAR	4.4	2–7

* Maximal sac diameter expansion, at the time of index ultrasound compared with earliest available post‐EVAR (or most recent revision) as demonstrated by CTA.

#### Case 4

An 80‐year‐old man at the time of index ultrasound, was referred for the US evaluation in the context of increasing sac size following original EVAR, 9 years earlier. His original procedure was complicated by SEWEL (sac expansion without endoleak), and he underwent endovascular revision 2 years later, followed by reduction in sac diameter. Interval increase in sac diameter was demonstrated at CTA over an 18‐month interval 3–5 years prior to index US; 60 x 49 mm to 67 x 59 mm, with subsequent increase to 70 x 70 mm over the following 3 years. Sac size was stable on multiple modalities for 12 months prior to index ultrasound. A suspected type II identified on prior CEUS was treated with angiographic sac puncture and glue embolisation one month prior to index ultrasound, though no definite endoleak was identified at that time. Of note, blood products were aspirated from the sac at the time of puncture.

**Fig. 4:**
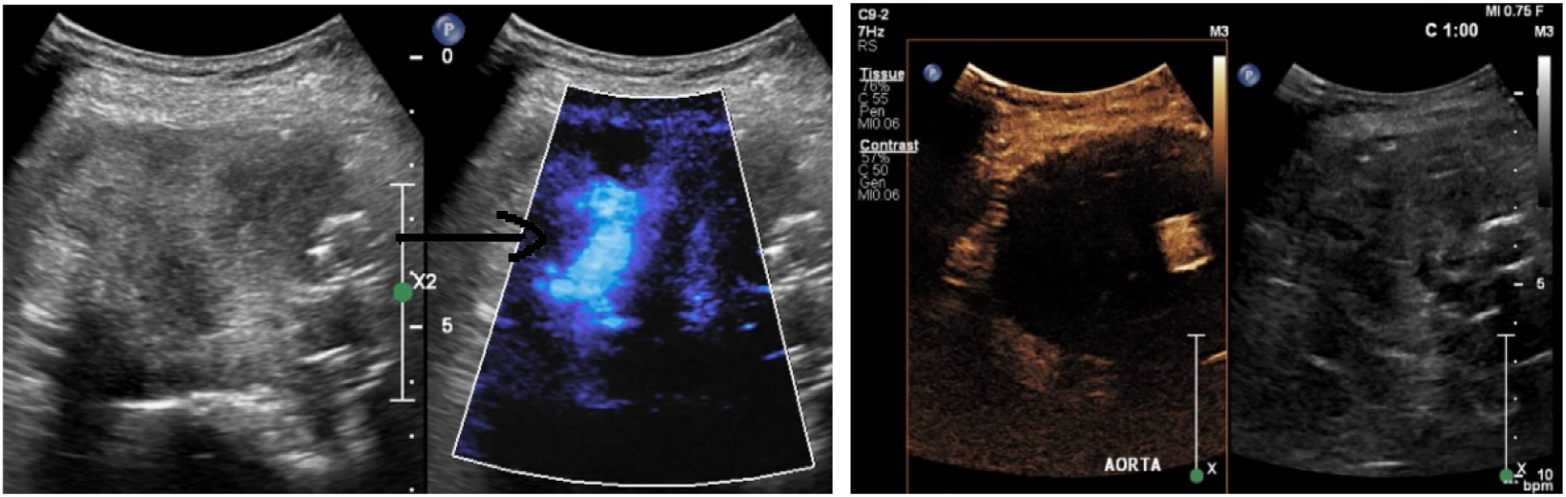
CDUS (left) showing flow on MFI within the excluded sac; CEUS (right) without demonstrable endoleak.

Index CDUS demonstrated a large amount of pulsatile material within the excluded sac; however, no endoleak was seen on CEUS (see Fig. 4).

No further management has been undertaken at this point, with ongoing surveillance planned.

#### Case 5

An 89‐year‐old man at the time of index ultrasound was referred for increasing sac size on CTA over a period of 13 months; from 81 × 85 mm to 85 × 90 mm. His original EVAR was performed 3 years prior to index ultrasound. No endoleak was demonstrated on CTA.

**Fig. 5:**
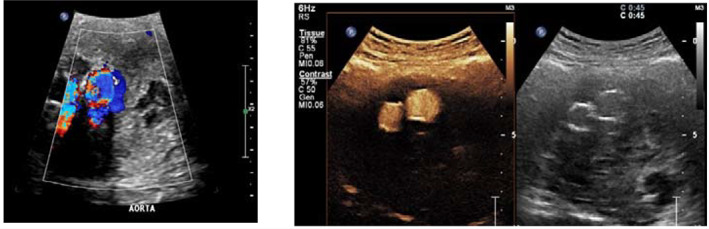
CDUS (left) showing flow within the excluded sac; CEUS (right) without demonstrable endoleak.

CDUS demonstrated movement within the excluded sac, but no endoleak was demonstrated on CEUS (see Fig. 5).

No further management at the time of writing, awaiting short‐term interval surveillance.

## Discussion

Each case in our series demonstrates a positive CDUS in the context of post‐EVAR surveillance, with extravascular doppler or MFI signal within the aneurysm sac, if followed by CEUS without demonstrable (Type I–IV) endoleak. Each case was preceded by aneurysm sac expansion on serial imaging, prompting the index ultrasound allowing for a diagnosis of SEWEL to be made.^12^

As an ultrasound‐based technique, flow on CDUS and MFI is sensitive in demonstrating fluid movement (i.e. blood flow) but is limited in its specificity.^13^ The movement of fluid within an excluded aneurysm sac as demonstrated on CDUS may occur as a result of either flowing blood in continuity with the arterial circulation (i.e. endoleak) or represent agitation of incarcerated fluid within the AAA sac (pseudoendoleak).

The absence of contrast material within the excluded sac on CEUS has a reportedly high negative‐predictive value for excluding endoleak, particularly type I and III.^10^ Our findings support that in the context of a positive CDUS surveillance study, CEUS is a useful adjunct test that can be performed with minimal risk, no iodinated contrast and no ionising radiation exposure. Specifically, in those patients with a positive CDUS, an expanding sac and a negative CT where pseudoendoleak is suspected.

Ultrasound is an operator‐dependent modality, and this also limits the generalisability of our findings. Each of our cases was either scanned primarily by a senior, specialist vascular sonographer, or in tandem with the same. Many EVAR patients are followed up with CDUS at our institution, where approximately 100 post‐EVAR CDUS are performed per year.

In conclusion, in our review of select post‐EVAR surveillance cases, CEUS is a useful discriminator between ‘false‐positive’ Doppler signal caused by agitated fluid (here termed pseudoendoleak) and true arterial blood flow in communication with the excluded EVAR sac (endoleak) particularly in the context of sac expansion with a negative CT and a positive CDUS. This is of particular clinical importance as it may avoid subsequent acute, potentially invasive diagnostic modalities such as sac puncture or premature EVAR revision.

## Data availability statement

The data that support the findings of this study are available on request from the corresponding author. The data are not publicly available due to privacy or ethical restrictions.
